# Isoproterenol Increases Uncoupling, Glycolysis, and Markers of Beiging in Mature 3T3-L1 Adipocytes

**DOI:** 10.1371/journal.pone.0138344

**Published:** 2015-09-21

**Authors:** Colette N. Miller, Jeong-Yeh Yang, Emily England, Amelia Yin, Clifton A. Baile, Srujana Rayalam

**Affiliations:** 1 Department of Animal and Dairy Sciences, University of Georgia, Athens, Georgia, United States of America; 2 Department of Foods and Nutrition, University of Georgia, Athens, Georgia, United States of America; 3 Complex Carbohydrate Research Center, University of Georgia, Athens, Georgia, United States of America; 4 Neuroscience Division, Biomedical and Health Sciences Institute, University of Georgia, Athens, Georgia, United States of America; 5 Pharmaceutical Sciences, Philadelphia College of Osteopathic Medicine–GA Campus, Suwanee, Georgia, United States of America; Faculty of Pharmacy, University of Lisbon, PORTUGAL

## Abstract

Beta-adrenergic activation stimulates uncoupling protein 1 (UCP1), enhancing metabolic rate. *In vitro*, most work has studied brown adipocytes, however, few have investigated more established adipocyte lines such as the murine 3T3-L1 line. To assess the effect of beta-adrenergic activation, mature 3T3-L1s were treated for 6 or 48 hours with or without isoproterenol (10 and 100 μM) following standard differentiation supplemented with thyroid hormone (T3; 1 nM). The highest dose of isoproterenol increased lipid content following 48 hours of treatment. This concentration enhanced UCP1 mRNA and protein expression. The increase in UCP1 following 48 hours of isoproterenol increased oxygen consumption rate. Further, coupling efficiency of the electron transport chain was disturbed and an enhancement of glycolytic rate was measured alongside this, indicating an attempt to meet the energy demands of the cell. Lastly, markers of beige adipocytes (protein content of CD137 and gene transcript of CITED1) were also found to be upregulated at 48 hours of isoproterenol treatment. This data indicates that mature 3T3-L1 adipocytes are responsive to isoproterenol and induce UCP1 expression and activity. Further, this finding provides a model for further pharmaceutical and nutraceutical investigation of UCP1 in 3T3-L1s.

## Introduction

Uncoupling proteins (UCPs) reside on the mitochondrial membranes and disrupt the proton gradient that drives the electron transport chain [1]. This results in the loss of protons as heat and subsequently causes increased fatty acid oxidation to meet the energy demands of the cell. Because of this property, the upregulation of UCPs have been targeted as a potential panacea for anti-obesity research [2]. Several isoforms of UCPs exist and further are found to populate differing tissue types, with UCP1 being the most highly researched due to its strong functional role in brown adipose tissue [3]. UCP1 can also be expressed in other adipocyte-types and depots, however its quantities and function vary depending on environmental conditions.

Physiologically, UCP1 responds to reductions in environmental temperature through the transient receptor potential family of ion channels (TRP V1, M8, A1) and subsequent sympathetic signaling [4]. All isoforms of the beta adrenergic receptors (ARs) exist on all adipose tissue types (white, beige, and brown) [5]. However, it is the β3 subclass that is expressed preferentially in adipocytes. Activation of the β3AR results in the upregulation of UCP1 expression via protein kinase A (PKA) and cAMP response element-binding protein (CREB) mediated transcription, however the other ARs may also contribute [6].

Isoproterenol is a non-specific AR agonist that upregulates UCP1 and thermogenesis. The vast majority of studies that have demonstrated this property has been performed *in vivo* and in *vitro* on brown adipocyte primary cells [7, 8]. Isoproterenol mediated UCP1 transcription has also been demonstrated in a variety of non-brown adipocyte cells including uterine smooth myocytes [9]. Further a variety of white adipocyte *in vitro* models have also shown upregulation of UCP1 following isoproterenol including mouse embryonic fibroblastic-derived adipocytes and primary white adipocytes from both rodents and humans [10–12].

The most commonly used adipocyte cell line and most characterized line is the murine 3T3-L1 line. Similarly to both white and brown adipocytes *in vivo*, 3T3-L1 cells express all ARs [13, 14]. Further, 3T3-L1 cells have shown responsiveness to isoproterenol and it has been widely used as a positive or negative control for the study of lipolysis in the line [15, 16]. Despite this well investigated response to isoproterenol, very little attention has been placed on UCP1 regulation in the 3T3-L1 line. In a recent study, a 4 hour treatment of isoproterenol following multiple adaptations to the standard differentiation protocol found an upregulation of UCP1 transcription [17]. No other work on the model has been published.

In the current study we sought to investigate if prolonged treatment of isoproterenol upregulates UCP1 transcription and activity using a single differentiation protocol in 3T3-L1 mature adipocytes. Using much lower doses of supplementary differentiation factors used in the Asano *et al* [17] study and longer treatment of isoproterenol, we hypothesized that we would see a similar upregulation of UCP1 in mature 3T3-L1 cells. Our study also sought out to further investigate if the increases in UCP1 levels and activity corresponded to changes in identified markers of beige adipocytes.

## Materials and Methods

### Culturing of 3T3-L1 Adipocytes

Mouse embryo 3T3-L1 preadipocytes were purchased from American Type Culture Collection (ATCC). Cells were maintained in DMEM F-12 supplemented with 10% bovine calf serum and pen-strep until confluent (Gibco; Grand Island, NY). To induce differentiation into mature adipocytes, cells were cultured in DMEM F-12 containing 10% fetal bovine serum (FBS) (Gibco), 5 μM dexamethasone (Sigma-Aldrich), 0.5 μg/mL insulin (Sigma-Aldrich), 0.5 mM isobutylmethylxanthine (Sigma-Aldrich), 1 μM rosiglitazone (Sigma-Aldrich), and 1 nM triiodothyronine (T3, Sigma-Aldrich) for 4 days. Cells were then switched to differentiation media II containing DMEM F-12 supplemented with 10% FBS, 0.5 ug/mL insulin, and 1 nM T3 for an additional 3 days. Following the 7 day differentiation protocol, mature 3T3-L1 adipocytes were treated with or without the beta-3 adrenergic agonist isoproterenol (Sigma-Aldrich) at doses of 10 or 100 μM for 6–48 hours for the following experiments. The doses of isoproterenol were selected from previous research on both brown and white adipocytes and have demonstrated UCP1 activation in such models [17–20].

### Cell Viability

3T3-L1 pre-adipocytes were seeded into a 96 well culture plate, grown to confluency, and differentiated as described above. Mature adipocytes were treated with 10 or 100 μM of isoproterenol for 6 or 48 hours. Immediately following treatment, cell viability was tested using the CellTiter 96® Aqueous One Solution Cell Proliferation Assay by Promega according to manufacturer’s protocols. Following 1 hour incubation, absorbance of metabolically active cells were measured at 490 nm in a FlexStation 3 plate reader (Molecular Devices).

### Lipid Quantification

AdipoRed™ reagent was used to quantify intracellular lipids in mature 3T3-L1 adipocytes according to manufacturer’s instructions (Lonza). Mature adipocytes were treated with or without varying doses of isoproterenol for 6 or 48 hours in a 96 well culture plate. Fluorescence was measured on a plate reader with excitation set at 485 nm and emission at 572 nm.

### Quantitative Real-Time Polymerase Chain Reaction

mRNA was isolated from mature 3T3-L1 adipocytes using QIAshredder and RNeasy mini-kit (Qiagen) after treatment for 6 and 48 hours with 10 or 100 μM isoproterenol. cDNA was synthesized using the High Capacity cDNA synthesis kit by Life Technologies according to manufacturer’s protocol. For qPCR 4 ng of cDNA was combined with DEPC-treated water, 1.6 uL of qPCR primers, and 10 uL of Sybr Green. qPCR was performed on the Applied Biosystems 7900 System. Table 1 details the primer sequences for genes related to thermogenesis and white and beige adipocyte markers. Primers were designed using the National Center for Biotechnology Information data base and purchased from Integrative Diagnostic Technologies.

**Table 1:** Primers used for Gene Expression Assays.

Classification	Gene Symbol	Sense	Anti-Sense
**Endogenous**	ACTB	ACCTTCCAGCAGATGTGGAT	TAGAAGCACTTGCGGTGCACGA
**control**	GAPDH	AACAGCAACTCCCACTCTTC	CCTGTTGCTGTAGCCGTATT
**Thermogenesis**	UCP1	CCTGGCAGATATCATCACCTTC	TGGTCCCTAGGACCTTTAT
	PRDM16	ACCTGCCACAGCAAAGAA	CCATCCAAGCAGAGAAGTAGAC
**Beige Markers**	CD137	GAGGTCAGAAGAGAAAGGGTTG	GTAGAGGACCCAGGTTTGATTC
	CITED1	AACCTTGGAGTGAAGGATCGC	GTAGGAGAGCCTATTGGAGATGT
	HOXC8	TAGTGTTGGCGGAGGATTTAC	TTGATCCGCGCCGTATTT
	HOXC9	TTGATCCGCGCCGTATTT	ACAGAACAATCAAGGCAGGT

The above primers were designed using the PubMed database and IDT, Inc for qPCR. For the purpose of dCt calculations, ACTB was chosen as a better endogenous control. Abbreviations: beta actin (ACTB), glyceraldehyde 3-phosphatase dehydrogenase (GAPDH), cluster of differentiation 137 (CD137), Cbp/p300-interacting transactivator 1 (CITED1), homeobox protein C8 (HOXC8), homeobox protein C9 (HOXC9), uncoupling protein 1 (UCP1), PR domain containing 16 (PRDM16).

### Immunocytochemistry

3T3-L1 pre-adipocytes were plated into glass slides and differentiated as described previously. 6–48 hours prior to fixation in 4% paraformaldehyde (PFA) cells were treated with or without isoproterenol (10 or 100 μM). For intracellular staining, cells were permeabilized with 0.1% triton x-100, 1% PVP in a PBS blocking solution containing 4% normal goat serum. Primary antibodies used were the beige marker CD137 (1:20; R&D Systems; #AF937) or UCP1 (1:50; Abcam; #ab10983). Primary antibodies were detected using a fluorescently conjugated secondary antibody, Alexa Flour 488 (1:500; Life Technologies; #A-11008). Lastly, DAPI (Life Technologies; #D1306) was used to visualize nuclei. Cells plated on slides were imaged on the IX81 with Disc-Spinning Unit (Olympus America, Inc.) using SlideBook Software (Intelligent Imaging Innovations, Inc.).

### Oxygen Consumption Rate

Premature 3T3-L1 adipocytes were seeded into the XFe24 Microplates by Seahorse Bioscience in a density of 20,000 cells/well. Cells were grown to confluence and differentiated into mature adipocytes following the protocol as described above. Mature adipocytes were treated with 10 or 100 μM isoproterenol for 6 and 48 hours prior to the start of the Seahorse XF Cell Mito Stress Test kit. Cells were washed 3 times and incubated in non-buffered DMEM supplemented with 25 mM glucose and 1 mM sodium pyruvate for 1 hour. The concentration of compounds used to determine oxygen consumption rate (OCR) included 1.5 μM oligomycin, 0.75 μM carbonyl cyanide-p-trifluoromethoxyphenylhydrazone (FCCP), 1 μM antimycin A and rotenone. The bioenergetics profile that is provided by the Seahorse Cell Mito Stress Test kit was determined by adjusting the values to the antimycin/rotenone treatment, which permits a more focused assay on the proton leakage. Coupling efficiency was determined by calculating the percentage of OCR immediately following the oligomycin treatment with the final baseline value.

### Extracellular Acidification Rate

Glycolytic rate was determined by monitoring extracellular pH induced by the change in lactate (extracellular acidification rate; ECAR). The ECAR assay was run in congruence with OCR using the conditions as described previously. Similarly to OCR, ECAR was adjusted to the values obtained during the antimycin/rotenone treatment. To determine the metabolic switch, the influence of isoproterenol on glycolysis and oxidative phosphorylation, basal OCR was plotted against basal ECAR rates. Lastly, to determine the effect of the uncoupling challenge during the assay on glycolytic rate, coupling efficiency was plotted against the first post-oligomycin ECAR level.

### Statistics

Between group differences were determined using t-tests (STATISTICA version 7.0). At least 3 biological replicates were performed for each assay and significance was set at p<0.05 for all tests.

## Results

### Cell Viability and Lipid Quantification

**Fig 1:**
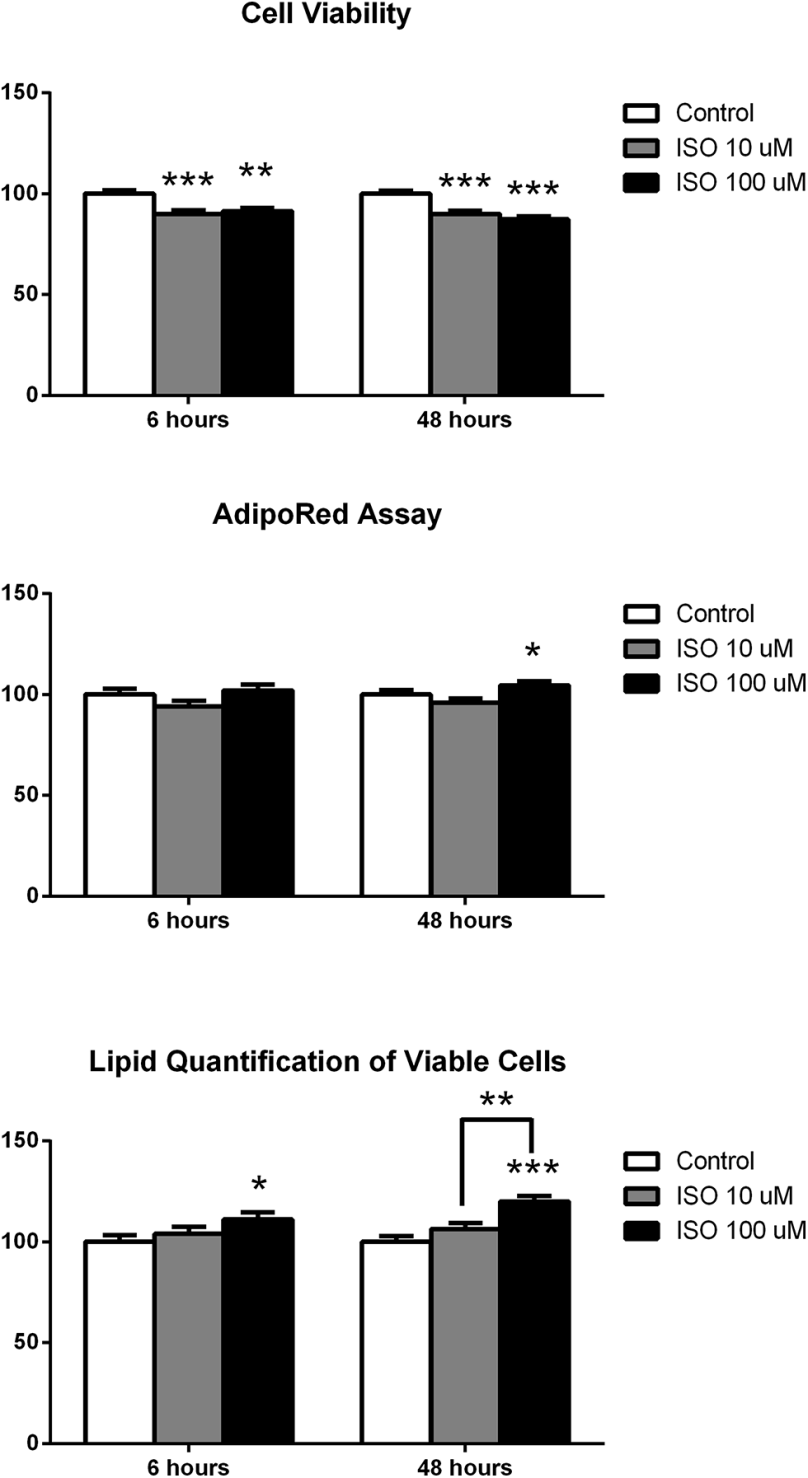
Cell Viability and Lipid Quantification of Mature Adipocytes Following Isoproterenol Treatment. Following 6 or 48 hours of isoproterenol treatment, cells were incubated with the Promega Cell Viability Reagent or the Lonza AdipoRed assay reagent and were read on a microplate reader. Each treatment contained 8 biological replicates. Data is represented as an adjusted percentage of control. Statistics were performed using t-tests. Statistical differences between control and treatment, unless otherwise designated, are indicated with * p<0.05, ** p<0.01, and *** p<0.001.

Cell viability was impacted by isoproterenol treatment at 6 hours and 48 hours (Fig 1A). Both doses of isoproterenol decreased the concentration of viable cells compared to control at both time points (p<0.01). In the unadjusted AdipoRed assay 6 hours of isoproterenol treatment proved to be insignificant in the amount of lipid determined, however a significant difference was measured between isoproterenol doses following 48 hours of treatment (p<0.01) (Fig 1B). Following adjustment for viable cells, isoproterenol treatment for 48 hours resulted in increased adjusted lipid content in a dose dependent fashion with the highest dose having the largest amount of lipid in viable cells (p<0.01) (Fig 1C). Six hours of the 100 μM isoproterenol treatment also increased the adjusted lipid content of mature 3T3-L1 adipocytes (p<0.05) (Fig 1C).

### Quantitative PCR

**Fig 2:**
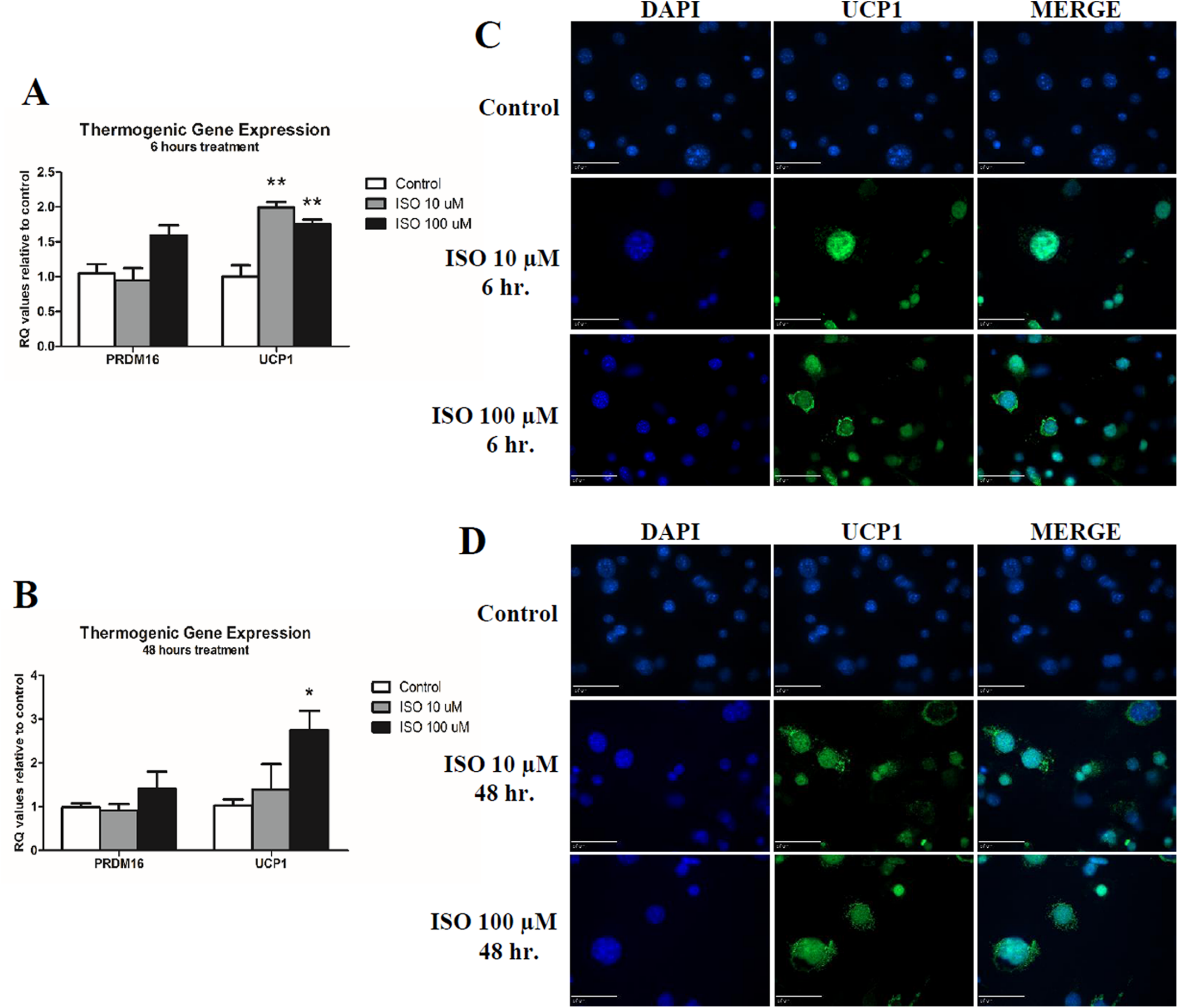
Quantitative PCR Analysis and Immunofluoresence of Thermogenic Markers in Mature Adipocytes. Mature 3T3-L1s were treated with either 10 or 100 μM isoproterenol for 6 and 48 hours before isolation of mRNA for qPCR (A & B respectively). Three biological replicates and technical replicates were used and data were normalized to β-Actin and control (no treatment). Statistics were performed using t-tests. Statistical differences between control and treatment, unless otherwise designated, are indicated with * p<0.05 and ** p<0.01. For immunoflouresence, cells were co-stained with anti-UCP1 in green following 6 or 48 hours of isoproterenol treatment (C & D respectively). Nuclear staining was performed using DAPI shown in blue. Scale bar equals 50 μM distance.

Following 6 hours, both doses of isoproterenol increased UCP1 mRNA compared to control (p<0.01) (Fig 2A). UCP1 expression was also increased following 48 hours of the 100 μM isoproterenol dose compared to control (p<0.05) (Fig 2B). No group differences were measured in PRDM16 expression at both time points (Fig 2A and 2B).

**Fig 3:**
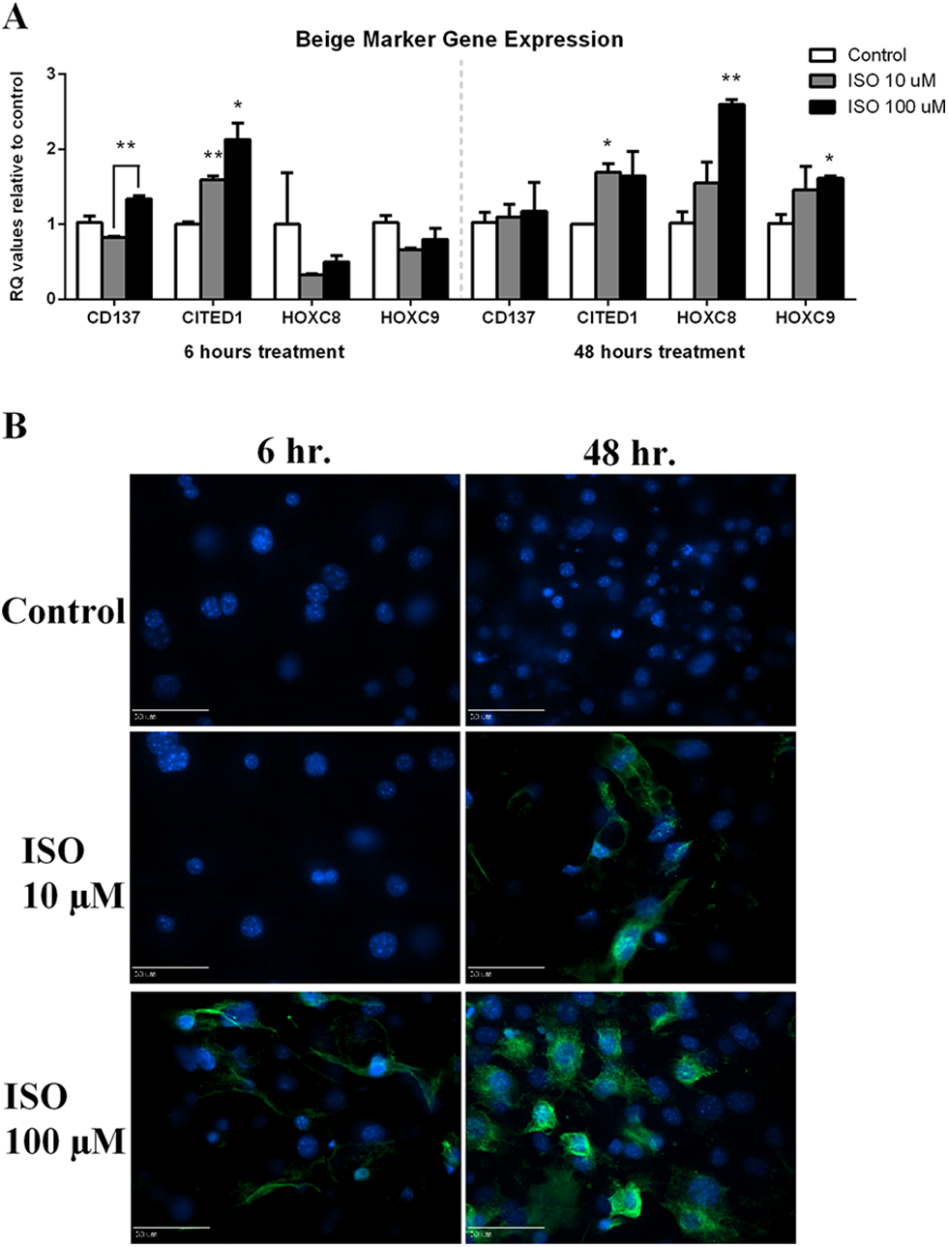
Expression and Immunofluoresence Staining of Beige Adipocyte Markers. Mature 3T3-L1s were treated with either 10 or 100 μM isoproterenol for 6 and 48 hours before isolation of mRNA for qPCR (A). Three biological replicates and technical replicates were used and data were normalized to β-Actin and control (no treatment). Statistics were performed using t-tests. Statistical differences between control and treatment, unless otherwise designated, are indicated with ** p<0.01. For immunoflouresence, cells were co-stained with anti-CD137 in green following 6 or 48 hours of isoproterenol treatment (B). Nuclear staining was performed using DAPI shown in blue. Scale bar equals 50 μM distance.

Expression of a genes that are commonly upregulated when cells have higher thermogenic activity and may indicate properties of beige adipocytes were additionally measured. CD137 expression was increased with the high dose, 100 μM isoproterenol treatment compared to control at the 6 hours assessment (p<0.01) (Fig 3A). Following 48 hours of treatment, there were no differences in CD137 expression. Both isoproterenol doses had higher expression of Cited1 compared to control following 6 hours (p<0.05) (Fig 3A). While no difference in Cited1 expression between control and the highest isoproterenol dose was measured following 48 hours of treatment, an individual t-test between the 10 μM dose and control was significant (p<0.01).

Lastly, additional genes were selected as they also are indicative of beige adipocytes but are not entirely a specific marker. Isoproterenol treatment for 6 hours failed to changes levels of HoxC8 or HoxC9 mRNA (Fig 3A). Following 48 hours of treatment, HoxC8 was increased in 100 μM isoproterenol treatment compared to control (p<0.01) (Fig 3A). Between treatment difference in HoxC8 expression neared significance at p = 0.06. A significant difference between control and the 100 μM isoproterenol treatment was measured in HoxC9 mRNA (p<0.05) (Fig 3A).

### Immunocytochemistry

UCP1 antibody staining was seen following both 6 and 48 hours of isoproterenol treatment, with no apparent effect of time or dose on the staining (Fig 2C and 2D). Both a time and dose dependent response was observed following antibody staining for the thermogenic adipocyte marker CD137 (Fig 3B).

### Oxygen Consumption Rate

**Fig 4:**
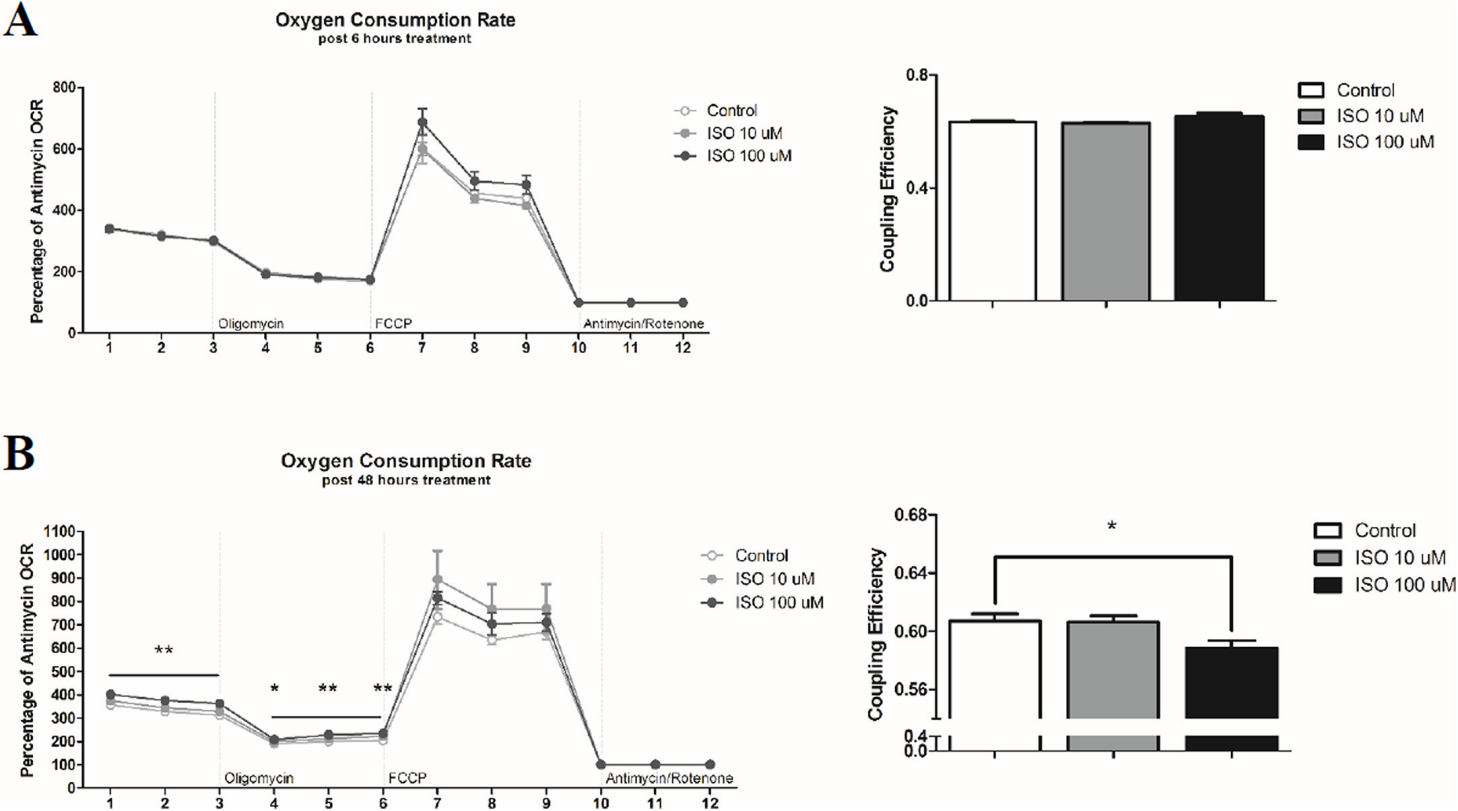
Oxygen Consumption Rate Following Isoproterenol Treatment. The Seahorse XF Cell Mito Stress Test was performed on mature 3T3-L1 adipocytes following a 6 (A) or 48 hour (B) treatment with varying doses of isoproterenol (ISO). The last of 3 measurements was used to determine percentage of change in oxygen consumption rate (OCR) within groups (n = 6 per treatment). Coupling efficiency was determined by calculating the percentage of OCR following oligomycin treatment from baseline. Statistical differences compared to control are indicated with * p<0.05, ** p<0.01, and *** p<0.001.

Because our study was interested in investigating changes in coupling efficiency and proton leak, the bioenergetics profile measured by the Seahorse Cell Mito Stress Test was adjusted to the mean of the final 3 measurements following antimycin and rotenone treatment. Following 6 hours of isoproterenol treatment, no differences were measured in either the adjusted OCR or coupling efficiency (Fig 4A). Differences between treatments became apparent following 48 hours of beta-agonist activation (Fig 4B). The baseline adjusted OCR was highest in cells treated with 100 μM isoproterenol compared to the 10 μM dosage and control at all three measurement points (p<0.01 compared to control and p<0.05 compared to 10 μM treatment). Following oligomycin treatment, which results in chemical-induced uncoupling, the 100 μM isoproterenol treatment group had increased OCR compared to control at all 3 measurement time points (p<0.05). Lastly, coupling efficiency was reduced in cells treated with 100 μM isoproterenol compared to both the 10 μM dose and control (p<0.05).

### Extracellular Acidification Rate

**Fig 5:**
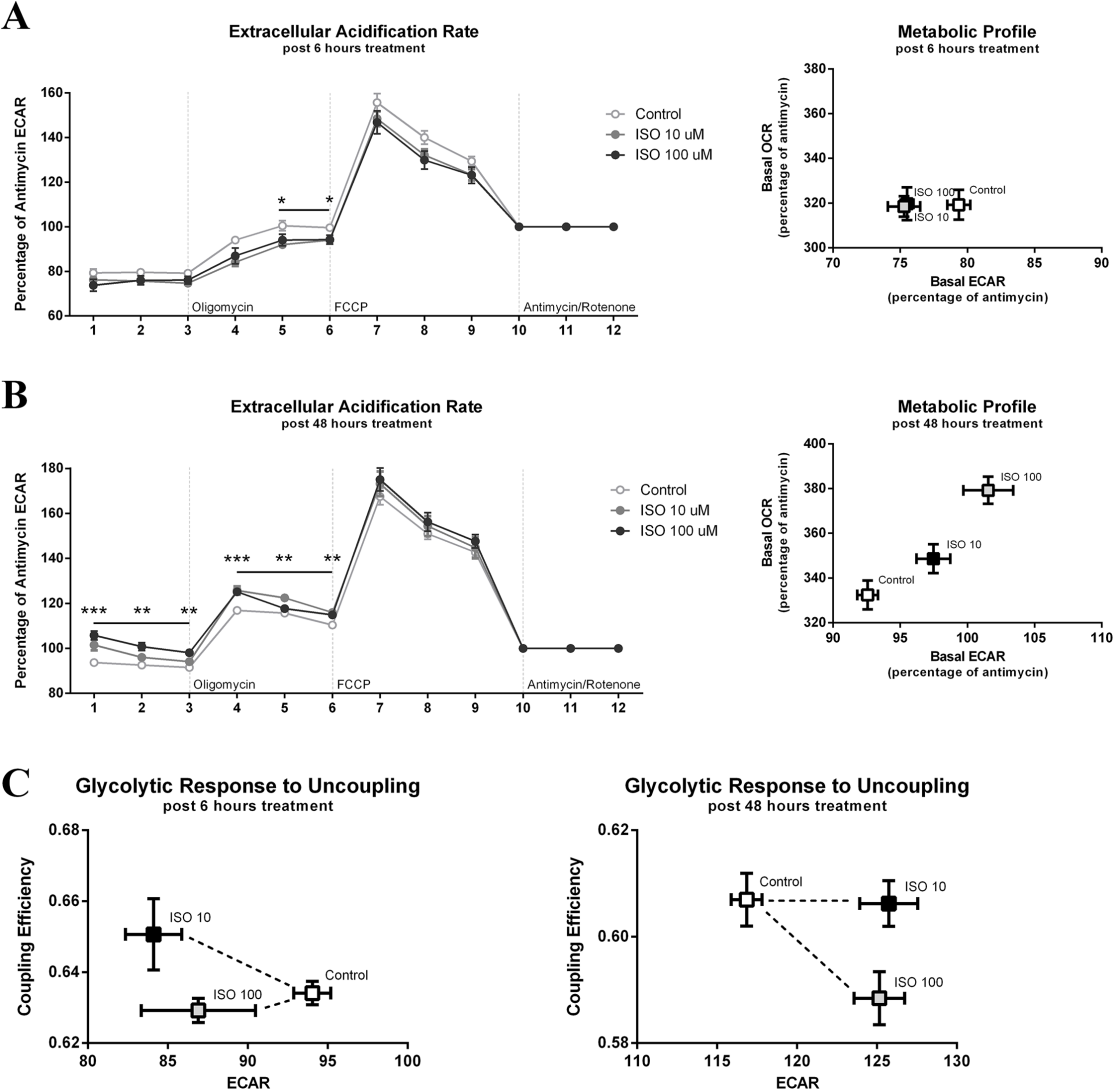
Extracellular Acidification Rate Following Isoproterenol Treatment. The Seahorse XF Cell Mito Stress Test was performed on mature 3T3-L1 adipocytes following a 6 (A) or 48 hour (B) treatment with varying doses of isoproterenol (ISO). The last of 3 measurements was used to determine percentage of change in extracellular acidification rate (ECAR) within groups (n = 6 per treatment) and provides a measurement of glycolysis. The metabolic profile for both 6 (A) and 48 hours (B) was determined by plotting ECAR to OCR. The relationship between coupling efficiency with glycolysis (ECAR) was visualized following 6 and 48 hours of isoproterenol treatment by plotting both points against each other (C). Statistical differences compared to control are indicated with * p<0.05, ** p<0.01, and *** p<0.001.

ECAR was used to determine changes in glycolytic rate. Acute exposure (6 hours) of isoproterenol treatment failed to change basal rates of glycolysis, however a slight, but significant reduction in glycolysis was measured following oligomycin treatment in the cells treated with both doses of isoproterenol (p<0.05) (Fig 5A). No differences in ECAR were found between isoproterenol doses. Further, the relationship between glycolysis (ECAR) and oxidative phosphorylation (OCR) was additionally plotted. No apparent association was seen between ECAR and OCR levels, suggesting that 6 hours of isoproterenol treatment dose not result in any shifts of the metabolic profile in 3T3-L1s.

Following 48 hours of 100 μM isoproterenol treatment in mature adipocytes, a significant increase in basal levels of glycolysis was measured at all three measurements (p<0.01) (Fig 5B). This glycolytic increase induced by 100 μM isoproterenol treatment was also found following the oligomycin, stimulated-proton leak phase (p<0.01). Further, the 10 μM isoproterenol dose also increased glycolysis during all three measurements following oligomycin treatment compared to non-treated cells (p<0.01). Plotting basal ECAR over OCR resulted in a clear positive relationship between isoproterenol treatment, and ECAR and OCR rates. This suggests that isoproterenol increases both glycolysis and oxidative phosphorylation in mature 3T3-L1 adipocytes, indicating a more metabolically active cell.

Lastly, ECAR was plotted against the coupling efficiency level at both 6 and 48 hours of isoproterenol treatment. No clear effect of isoproterenol was seen after 6 hours beyond the aforementioned reduction in ECAR (Fig 5C). However, following 48 hours of 100 μM isoproterenol treatment a negative relationship between coupling efficiency and ECAR emerged. This suggests that as coupling efficiency reduces in high dose-treated cells, glycolysis increases.

## Discussion

In the current study it was found that both UCP1 transcript and protein was upregulated within 6 hours of isoproterenol treatment in mature 3T3-L1 adipocytes. While UCP1 levels were increased at the 6 hour time point, it was not until 48 hours of isoproterenol treatment that enhanced uncoupling of the electron transport chain and increases in glycolytic rate could be measured.

The expression patterns of ARs in 3T3-L1s have been reported to change throughout the differentiation process [14, 21]. Specifically, in undifferentiated, immature 3T3-L1s there are no differences between β1 and β2 concentrations. Following differentiation, mature 3T3-L1s demonstrate a clear preference for the β2 subclass (95:5) [14]. Manipulation of the components of the differentiation cocktail can enhance and repress AR expression accordingly and impact differentiation. Because of the adaptations of the AR profile during the differentiation process, it is important to investigate the response to the non-specific AR agonist, isoproterenol, in a variety of 3T3-L1 models.

Asano *et al* demonstrated that changes in the differentiation cocktail, including the removal of IBMX and addition of both T3 and rosiglitazone during the differentiation process greatly impacted the responsiveness to isoproterenol [17]. They report that the addition of all three are necessary for the proper upregulation of UCP1 with isoproterenol. Rosiglitazone can reverse the effects of AR antagonists [22]. Further, T3 also demonstrates the ability to upregulate UCP1 via the *cis*-thyroid response element on the UCP1 promoter and has been implicated as a required factor necessary for the AR-induced stimulation of UCP1 [23, 24]. Thus, it appears that both T3 and rosiglitazone are potent sensitizers to ARs. In a cell such as a white adipocyte, the addition of both during the differentiation process appears necessary to achieve the isoproterenol-UCP1 response [17].

In the present study a low dose of T3 was used during differentiation, which is the lowest dose used in brown adipocyte differentiation protocols [25]. With this very low dose (1 nM) of T3, we were able to demonstrate an upregulation of UCP1 transcript at 6 hours of isoproterenol treatment. This increase was found at both doses of isoproterenol, 10 and 100 μM, and thus we replicate the results of Asano *et al* [17]. The aforementioned study however failed to measure changes in metabolic activity and electron transport efficiency following isoproterenol treatment. Primary white adipocytes have previously demonstrated the ability to upregulate oxygen consumption rate and uncoupling with high dose isoproterenol in a very acute fashion (30 minutes) [10]. Thus, we tested the potential of the isoproterenol-primed 3T3-L1 to the cellular mitochondrial stress assay by Seahorse Bioscience. At the 6 hour time point, both doses of isoproterenol failed to impact any of the respiration tests. Thus, we report that while there is an upregulation of UCP1 transcript and protein, it not enough to induce functional changes in the 3T3-L1 despite differentiational priming and differing AR agonist dosage.

Prolonged exposure to isoproterenol (48 hours) using the priming differentiation protocol did induce changes in oxygen consumption rate during baseline measurements and during the inhibitory phase following oligomycin treatment. Further, as the oligomycin phase demonstrated, our high dose isoproterenol treatment (100 μM) did induce reductions in coupling of the electron transport chain, and thus suggests an increase in UCP1 activity in these cells.

The presence of uncouplers, including UCP1, promotes a variety of metabolic changes in the cell. While fatty acid oxidation in response to UCP1 activation is well demonstrated, increases in glycolytic rate has also been hypothesized due reduced efficiency of ATP formation [26, 27]. Following 48 hours of isoproterenol treatment an increase in ECAR which measures changes in extracellular pH as a response to lactate formation was found. Particularly within the high dose, 100 μM treatment, isoproterenol resulted in a significant shift to an anaerobic/glycolytic increase as coupling efficiency dropped. Overall, this data indicates that isoproterenol induces an upregulation of UCP1 that results in changes in a variety of metabolic pathways in mature 3T3-L1 adipocytes.

The AdipoRed assay is often used as a proxy for lipogenesis and was used in the current study to reflect changes in intracellular lipid concentrations. Isoproterenol rapidly induces lipolysis, within 3–4 minutes, in mature adipocytes via a protein kinase A and hormone sensitive lipase mechanism [28]. Additional research has also shown that AMPK may also play an important role in this early isoproterenol response [29]. In contrast, data from the current study showed an increase in intracellular lipid content following 48 hours of 100 μM isoproterenol treatment. As other studies have shown, longer exposures to isoproterenol (16 hours) results in a downregulation of adipose triglyceride lipase, an important catalase for the breakdown of triglycerides [30]. However it is also known that isoproterenol induces insulin resistance in 3T3-L1 cells, mediated through the pro-inflammatory cytokine suppressor of cytokine signaling 3 (SOCS3) [31]. Thus, our finding of enhanced lipid content in addition to enhanced uncoupling is not entirely clear. Chronic beta-adrenergic activation (7 days) *in vivo* results in increased lipogenesis and lipolysis in brown, white, and beige adipose tissues [32]. However, because the previous *in vitro* work has not extended isoproterenol treatment to the length of our current study, it is difficult to identify the specific mechanisms that led to the upregulated lipid content we observed and thus indicates the need for additional research.

Lastly, due to the conclusions of Asano *et al* and the known implications of UCP1 upregulation in white adipocytes, we investigated changes in the beige adipocyte markers CD137 and CITED1 [17, 33]. An increase in CITED1 expression was previously reported, however was not observed in the T3, rosiglitazone, and IBMX positive differentiation protocol that was necessary for UCP1 expression [17]. In the current study, CITED1 and the CD137 transcript and protein was found to be increased following isoproterenol alongside increases in UCP1. At the dose used in Asano *et al* (10 μM) we replicated the increases in CITED1 however no noticeable changes in CD137 was measured at the similar time point. The higher dose used in this study (100 μM) did induce an upregulation of CD137 following 6 hours of treatment, both transcript and protein, however the upregulation of the transcript was lost at 48 hours. CITED1 upregulation was also lost under these conditions, however this may have been due to a large standard error as the 10 μM isoproterenol dose increased CITED1 at 48 hours. Similarly, the non-specific beige markers HOXC8 and HOXC9 were upregulated at 48 hours of 100 μM isoproterenol.

Taking into account the two studies, Asano *et al* and ours, we conclude that 3T3-L1s have the ability to induce UCP1 and markers of beige adipocytes under specific environmental conditions. Data from our study adds to the growing evidence that white adipocytes are responsive to beta agonists and can upregulate UCP1 concentrations that lead to measurable changes in cellular metabolic pathways. Findings of our study renders further investigation of both UCP1 activation and potential beiging properties of 3T3-L1 adipocytes. Lastly, as this model continues to develop, we provide evidence that the 3T3-L1 line may be used in future screenings to determine efficacy of anti-obesity pharmaceutical and nutraceutical compounds to increase metabolic rate.
